# Beware of reflectance confocal microscopy artifacts when searching hyphae in acral skin – Reply^☆^^☆☆^

**DOI:** 10.1016/j.abd.2019.04.014

**Published:** 2019-12-30

**Authors:** John Verrinder Veasey

**Affiliations:** Infectious Dermatoses Sector, Clínica de Dermatologia, Santa Casa de Misericórdia de São Paulo, São Paulo, SP, Brazil

Dear Editor,

I would like to thank Cinotti et al. for their interest in our paper about tinea nigra's findings at confocal microscopy, as well as the opportunity offered by the editors of this journal to answer the points raised by the authors in the article “Beware of reflectance confocal microscopy artifacts when searching hyphae in acral skin”.

The etiological agent of tinea nigra, *Hortaea wernekii*, is a dematiaceous geophilic fungus. The dermoscopic features of this disease consist of a fibrillar hyperchromic pattern, and the direct mycological examination (DME) presents short and thick dematiaceous hyphae, corresponding to the described dermoscopic findings.^1^, ^2^ Other cases seen in our service with fungal culture isolating *H. wernekii* are compatible with these findings (Fig. 1).

**Figure 1:**
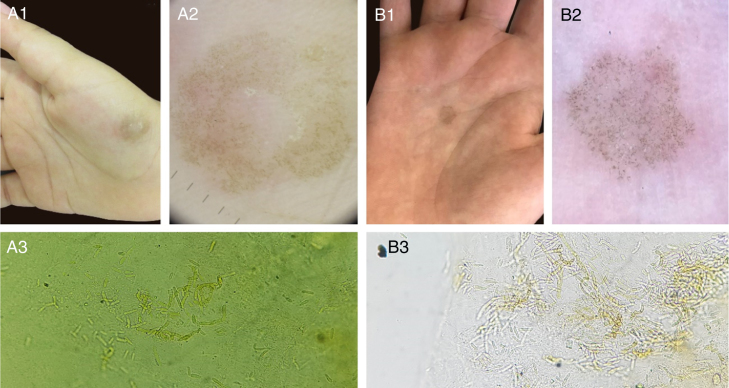
Two cases of tinea nigra with classic clinical presentation (A1, B1). Dermoscopy of both cases, with short hyperchromic linear structures in the epidermis (A2, B2). Direct mycological examination (KOH 20%), with short dematiaceous septate hyphae (A3 x200, B3 x400).

Such facts had led us to believe that the images identified by reflectance confocal microscopy (RCM) in our article would be compatible with the hyphae of *H. wernekii* parasitizing the epidermis. It is noteworthy that the morphology of short thick structures presented at dermoscopic examination and DME were compatible with those evidenced by the RCM.^3^

As for the images presented in the response correspondence, composed of few thin and elongated septate hyphae at the surface of the epidermis, I would like to point out that in human microbiome studies there are evidence of filamentous fungi in the skin of healthy patients.^4^, ^5^ Such agents, when provoking superficial mycoses, present at the DME fine and long hyphae (Fig. 2), a morphology that has also been described in RCM analyzes.^6^, ^7^, ^8^, ^9^ An important fact in Cinotti et al. article was the lack of fungal culture of the specimen evidenced by RCM images, making it impossible to confirm that the hyphae visualized were of *H. wernekii*. I believe that these facts should be taken into account when finding thin and long hyphae in the analysis of tinea nigra images, since the other tests performed for its diagnosis usually do not present these thin structures. Unfortunately, it is not possible to evaluate by the RCM if the hyphae presented in the cases are hyaline or dematiaceous, since both melanin and the cellular wall of the fungi present white color by the RCM examination.

**Figure 2:**
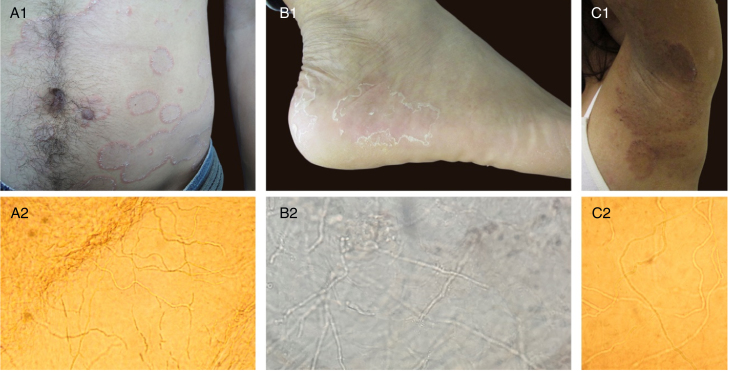
A1, B1, C1: clinical aspect of superficial mycoses. A2, B2, C2: direct mycological examinations (KOH 20%) corresponding to clinical presentations, presenting in all cases thin and long hyaline septated hyphae (x100, x400, x200, respectively).

Finally, it cannot be denied that the use of RCM for the analysis of tinea nigra presents various pitfalls, either due to the presence of filamentous fungal structures that could erroneously be considered compatible with *H. wernekii*, or to the presence of epidermal artifacts that could be considered as fungal structures. Further studies are required to help us elucidate the structures of this dermatosis.

## Financial support

None declared.

## Author's contribution

John Verrinder Veasey: Approval of the final version of the manuscript; elaboration and writing of the manuscript; collection, analysis, and interpretation of data; intellectual participation in the propaedeutic and/or therapeutic conduct of the studied cased; critical review of the literature; critical review of the manuscript.

## Conflicts of interest

None declared.
